# Molecular Epidemiology of Rabies Viruses Circulating in Two Rabies Endemic Provinces of Laos, 2011–2012: Regional Diversity in Southeast Asia

**DOI:** 10.1371/journal.pntd.0003645

**Published:** 2015-03-31

**Authors:** Kamruddin Ahmed, Phouvong Phommachanh, Phengphet Vorachith, Takashi Matsumoto, Pheophet Lamaningao, Daisuke Mori, Minako Takaki, Bounlom Douangngeun, Bounkhouang Khambounheuang, Akira Nishizono

**Affiliations:** 1 Department of Microbiology, Faculty of Medicine, Oita University, Yufu, Japan; 2 Research Promotion Institute, Oita University, Yufu, Japan; 3 National Animal Disease Diagnostic Laboratory, Vientiane capital, Laos; 4 Department of Public Health, Kansai Medical University, Osaka, Japan; 5 National Animal Health Center, Vientiane capital, Laos; 6 Department of Livestock and Fisheries, Ministry of Agriculture and Forestry, Vientiane capital, Laos; The Global Alliance for Rabies Control, UNITED STATES

## Abstract

**Background:**

Although rabies is endemic in Laos, genetic characterization of the viruses in this country is limited. There are growing concerns that development in the region may have increased transport of dog through Laos for regional dog meat consumption, and that this may cause spillover of the viruses from dogs brought here from other countries. This study was therefore undertaken to evaluate the current rabies situation and the genetic characteristics of rabies viruses currently circulating in Laos.

**Methods:**

We determined the rate of rabies-positive samples by analyzing data from animal samples submitted to the Lao Ministry of Agriculture and Forestry’s National Animal Health Centre rabies laboratory from 2004 through 2011. Twenty-three rabies-positive samples were used for viral genetic characterization. Full genome sequencing was performed on two rabies viruses.

**Results:**

Rabies-positive samples increased substantially from 40.5% in 2004 to 60.2% in 2009 and continued at this level during the study period. More than 99% of the samples were from dogs, followed by cats and monkeys. Phylogenetic analyses showed that three rabies virus lineages belonging to the Southeast Asian cluster are currently circulating in Laos; these are closely related to viruses from Thailand, Cambodia and Vietnam. Lineages of the circulating Laos rabies viruses diverged from common ancestors as recently as 44.2 years and as much as 55.3 years ago, indicating periodic virus invasions.

**Conclusion:**

There is an increasing trend of rabies in Laotian animals. Similar to other rabies-endemic countries, dogs are the main viral reservoir. Three viral lineages closely related to viruses from neighboring countries are currently circulating in Laos. Data provide evidence of periodic historic exchanges of the viruses with neighboring countries, but no recent invasion.

## Author Summary

Laos is a land-locked rabies-endemic country in Southeast Asia that is surrounded by five rabies-endemic countries. Thus, there is increasing concern that the epidemiology of rabies in Laos is influenced by infrastructure development and economic activities, including international transport of dogs for meat consumption. Studies on the epidemiology of rabies are limited in this country. Therefore, to gain further information about the epidemiology and the genetic characteristics of circulating rabies viruses, this study was conducted using samples submitted to the rabies Lao Ministry of Agriculture and Forestry’s National Animal Health Centre rabies laboratory. Out of 18 provinces, samples were submitted mainly from the capital Vientiane and Champasak province. Data from the period 2004 through 2011 showed a gradual increase in rabies-positive samples. Dogs were the main viral reservoir, and genetic analyses of samples collected from February 2011 through March 2012 showed that three viral lineages are currently circulating in the country. These rabies viruses are related to those of neighboring countries, indicating shared ancestry but no recent viral invasion.

## Background

Globally, an estimated 60,000 people die of rabies annually, and more than 31,000 of these deaths occur in Asia [1,2]. Among various Asian regions, the countries of the Association of South East Asian Nations (ASEAN) have been working towards substantial economic development. A call for elimination of rabies by 2020 highlights the political importance of rabies control [3]. Seven out of the 10 ASEAN member states are rabies-endemic: Cambodia, Indonesia, Laos, Myanmar, the Philippines, Thailand and Vietnam [3]. Among these countries, Laos has the lowest reported number of human deaths from rabies. However, the precise morbidity, mortality and molecular epidemiology of rabies in Laos is largely unknown. This is due to difficulty in collecting data and samples from remote areas of the country, and to the country’s modest data collection system at the centralized diagnostic facility. Survey of prevalence of canine rabies is still in its initial stages and limited to a small central part of Laos. The first reported phylogenetic study showed that Laotian rabies viruses are grouped with viruses from Thailand, Myanmar, Cambodia and Vietnam [4]. Combined analyses from geographic information system data showed that China is the likely source of the Asian rabies viruses, whereas individual migration event suggested that Cambodia may be a source of Asian rabies viruses transmission to China, Laos and Thailand [5].

The Laotian government vision is to convert the country from a ‘land-locked’ to ‘land-linked’ due to its strategic position in the ASEAN region. As a result, a number of bridges and new roads have been built to connect Laos to neighboring countries. Since ancient times, Laos has relied on its neighbors for trading. For historical, travel convenience and cultural reasons, Thailand has been a primary trading partner. The Mekong River region is well known for its culture of dog-meat consumption [6]. With growing economic prosperity and improved roads across this region, movement of people and allegedly, dogs for meat consumption has been increasing substantially. These factors may have influenced the dissemination and evolution of rabies viruses.

That rabies is a neglected tropical disease is exemplified by the fact that with the exception of Thailand, a full viral genome sequence has been unavailable across the rabies-endemic South East Asian countries. Rabies belongs to the genus *Lyssavirus* of the family *Rhabdoviridae*. It is a single-stranded, negative-sense RNA of approximately 12 kb that encodes five structural proteins: nucleoprotein (N), phosphoprotein (P), matric protein (M), glycoprotein (G), and RNA-dependent RNA polymerase (L). These genes are separated by intergenic regions of variable length. Phylogenetic classification of rabies viruses is based on specific genes, mostly from partial gene sequences, and thus may lead to inconsistent results. It is possible that phylogenetic classification using complete genome sequences, rather than from a partial sequence of a specific gene, would offer a more robust and comprehensive means of addressing the evolution, spread and genome-wide heterogeneity of viruses [7]. Complete genome sequencing of rabies viruses from these countries will also help explain the evolution of the viruses in Laos and the Mekong River region. Because only complete genome sequence can reveal the length, nucleotide substitution patterns, and variations in the start signal, stop signal and other motifs across the genes. As a result it can provide more information to compare and can generate reliable results on genome evolution. Therefore, this study was undertaken to quantify the current animal rabies occurrence in Laos and to complete a molecular characterization of the viruses in current circulation.

## Methods

### Ethics statement

The study was approved (No. 38/NECHR) by the National Ethics Committee for Health Research, Ministry of Health, Laos.

### Collection of data

To determine the prevalence of rabies, we analyzed data from samples submitted from various regions of Laos to the rabies laboratory of the National Animal Health Centre, Department of Fisheries and Livestock, Ministry of Agriculture and Forestry from January 2004 through December 2011. This is the only central animal rabies diagnostic laboratory in Laos. In Laos, dog and cat rabies are notifiable, but not wildlife rabies. For animal samples, the head of the suspected animal was submitted to the laboratory, and the brain was dissected by a trained technician. These animals had signs of rabies such as aggressive behavior, a tendency to bite and excessive salivation, with or without a history of biting humans and/or animals. There are no community veterinarians in Laos; samples from suspect animals are submitted mainly by the general public.

Brain sample tests are performed using a fluorescent antibody test (FAT) on crushed smears of hippocampus for a fee of 30,000 Kip (approximately US$ 3.70) and test results are provided on paper to the individual who submitted the sample. In emergency cases, such as when postexposure prophylaxis (PEP) is required, the person is informed of the results by phone.

### Sample collection for molecular characterization of rabies virus

**Fig 1:**
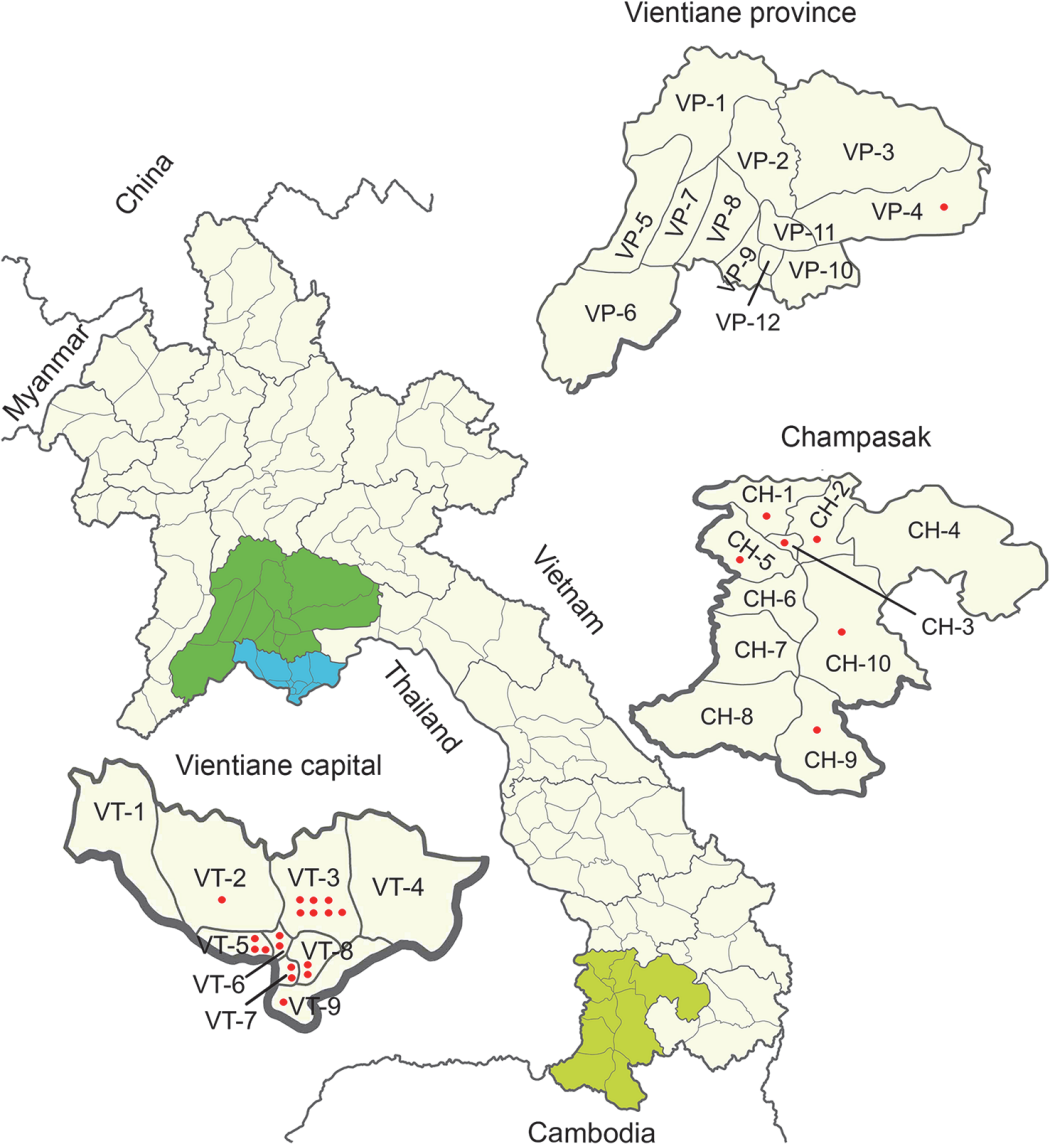
Map of Lao Peoples Democratic Republic. Map showing three provinces of Lao from where florescent antibody test positive rabies samples were collected. Samples collected from the districts of Vientiane capital (blue) are as follow: 7 from Xaythany (code VT-3), 3 from Sikhottabong (VT-5), 2 from Sisattanak (VT-7) and Saysettha (VT-8), and 1 from Hatsayfong (VT-9) and Naxaythong (VT-2). One sample was collected from each of the following districts of Champasak (light green): Sanasomeboune (CH-1), Bachieng (CH-2), Pakse (CH-3), Kong (CH-9), Phonthong (CH-5), and Pathoumphone (CH-10). One sample was collected from Meuan district of Vientiane province (green). Each red dot represents one rabies-positive sample. See S1 Table for more detailed information of the samples.

The samples submitted from Vientiane capital, Vientiane and Champasak provinces (Fig 1) to the rabies laboratory of National Animal Health Centre, during February 2011 through March 2012, were used in this study for molecular characterization (S1 Table).

### RNA extraction, RT-PCR and nucleotide sequencing

Total RNA was extracted from about 1 g of brain homogenate by using Trizol (Invitrogen, Carlsbad, CA, USA) according to the manufacturer’s instruction. Extracted RNA was stored at -30°C until further analyses.

Using random hexamer primers cDNA was synthesized by SuperScript III First-Strand Synthesis System (Invitrogen) according to the instructions of the manufacturer. The synthesized cDNA was diluted with DNase/RNase free water (Invitrogen) as a template. PCR was performed using TaKaRa ExTaq (Takara Bio Inc., Shiga, Japan). Nested PCR for nucleoprotein (N) gene was performed to detect the presence of rabies virus genome in the samples [8]. The PCR for N and glycoprotein (G) genes, and G-L intergenic region was performed [7]. Whole genome sequence of two strains, arbitrarily selected from Vientiane capital and Champasak samples was performed [7]. For 13 other strains only full-length N gene sequence could be performed. Cycle sequencing of the amplified product was performed using the BigDye Terminator v3.1 Cycle Sequencing Kit (Applied Biosystems, Foster city, CA, USA). The purified amplicons were sequenced using ABI-3130 Genetic sequencer (Applied Biosystems). All steps were done according to the manufacturer’s instruction. The 5′ or 3′-terminal end of the genome was amplified using SMART RACE cDNA Amplification Kit (Clontech Laboratories, Inc., Mountain View, CA, USA) according to the instructions of the manufacturer.

### Time-line evolutionary analysis

Evolutionary analysis was done using the full-length N gene. We inferred a Maximum Clade Credibility phylogenetic tree using the Bayesian Markov Chain Monte Carlo method available in the BEAST package, v1.6.1 [9]. The analysis utilized a relaxed (uncorrelated lognormal) molecular clock and GTR+Γ+I model of nucleotide substitution. The model was selected on the basis of Akaike Information Criterion using jModelTest software [10]. All chains were run for 60 million generations and sampled every 3000 steps. This resulted in an effective sample size of >521 for all estimated parameters. The posterior densities were calculated using with 10% burn-in and checked for convergence using Tracer, v.1.5.

### Sequence analyses and construction of whole genome phylogenetic tree

The nucleotide and amino acid sequences of genes and intergenic regions were compared among rabies viruses from Laos. The nucleotide sequences were used to construct the whole genome phylogenetic tree [7]. Multiple sequence alignment was done by ClustalW ver.2 then phytogenic tree was constructed with MEGA ver. 5 using the neighbor joining (NJ) method and the branching pattern was statistically evaluated by bootstrap analysis of 1000 replicates.

## Results

### Epidemiology of rabies

During this eight years period, number of submitted samples peaked in 2008 and gradually decreased (Table 1). Whereas number of rabies positive samples varied annually, peaked in 2009 and gradually decreased. However the percentage of rabies positive samples increased significantly from 40.5% in 2004 to 60.2% in 2009 and continued at this level.

**Table 1:** Yearly distribution of samples submitted from different animals for the laboratory diagnosis of rabies and their proportion positive for rabies.

**Year**	**Dog**	**Cat**	**Rat**	**Monkey**	**Rabbit**	**Total**
**2004**	66/162	0	0/1	0	0	66/163 (40.5%)
**2005**	84/167	0	0	0	0	84/167 (50.3%)
**2006**	87/179	0	0/1	0	0	87/180 (48.3%)
**2007**	64/151	0/1	0	0	0	64/152 (42.1%)
**2008**	102/198	1/1	0	0	0	103/199 (51.7%)
**2009**	105/174	3/5	0	1/1	0/1	109/181 (60.2%)
**2010**	72/117	0/1	0	0	0	72/118 (61.0%)
**2011**	60/101	0	0	0	0	60/101 (59.4%)
**Total**	640/1249	4/8	0/2	1/1	0/1	645/1261 (51.1%)

Annually, mean 157.6 {95% confidence interval (CI) 129.9–185.3} samples were submitted for rabies diagnosis, among them 80.6 (95% CI 65.3–95.9) samples were rabies positive i.e. 51.7% (95% CI 45.0–58.4%) of the submitted samples were rabies positive. Most of the submitted samples were from dogs 155.1 (95%CI 129.0–183.2), followed by cats 1.0 (95% CI 0–2.4), and monkeys 0.5 (95% CI 0–1.1). Likewise most of the rabies positive samples were also from dogs 80.0 (95%CI 65.5–94.4), followed by cats 0.5 (95%CI 0–1.4), and other animals 0.13 (95% CI 0–0.4). These represent that 99.2% of the rabies samples were from dogs followed by cats and monkeys (0.6% and 0.3%, respectively).

Province-wise (Table 2) data analyses showed that yearly submitted samples were mainly from Vientiane capital 115.1 (95% CI 90.2–140.0), followed by Champasak 25.4 (95% CI 14.3–36.4), Vientiane province 11.7 (95% CI 6.6–16.9), and other provinces 5.4 (95% CI 3.1–7.6). Samples from Vientiane capital represents 73.0% of the total submitted samples followed by Champasak (16.1%), Vientiane province (11.7%), and other provinces (3.4%). From other provinces sample number never exceeded more than six and usually only one per year. Samples were never received during this period from six of 18 provinces of Laos.

**Table 2:** Yearly distribution of province wise submitted samples for the laboratory diagnosis of rabies.

	Vientiane capital	Vientiane province	Chamapasak	Salavanh	Bolikhamxay	Xekong	Xiengkhouang	Savanhakhet	Luangprabang	Attapeua	Oudomxay	Luangnamtha	Total
2004	104	24	24	3	6	1	1						163
2005	135	14	16			1		1					167
2006	148	11	15		2	1	1		1	1			180
2007	122	13	14			1	1				1		152
2008	136	6	51	3	1		1		1				199
2009	133	4	39		2	1	2						181
2010	74	14	26			2	2						118
2011	69	8	18			1	1					4	101
Total	921	94	203	6	11	8	9	1	2	1	1	4	1261

### Time-line evolutionary analysis

The presence of rabies virus was confirmed in 21 of the 23 FAT positive samples by RT-PCR and partial nucleotide sequence of these amplicons. Lao9 and Lao19 samples from Vientiane capital were negative by RT-PCR. The full-length N gene sequence could be done in a total of 15 samples that were used in time-line evolutionary analysis. These samples were from Vientiane capital and Champasak provinces, the full-length N gene of the only sample from Vientiane province could not be amplified. The mean rate of nucleotide substitution estimated for the N gene was 2.5×10^4^ substitutions/site/year (95% HDP values 1.6–3.4×10^4^ substitutions/site/year). This rate is in agreement with previous studies [11].

**Fig 2:**
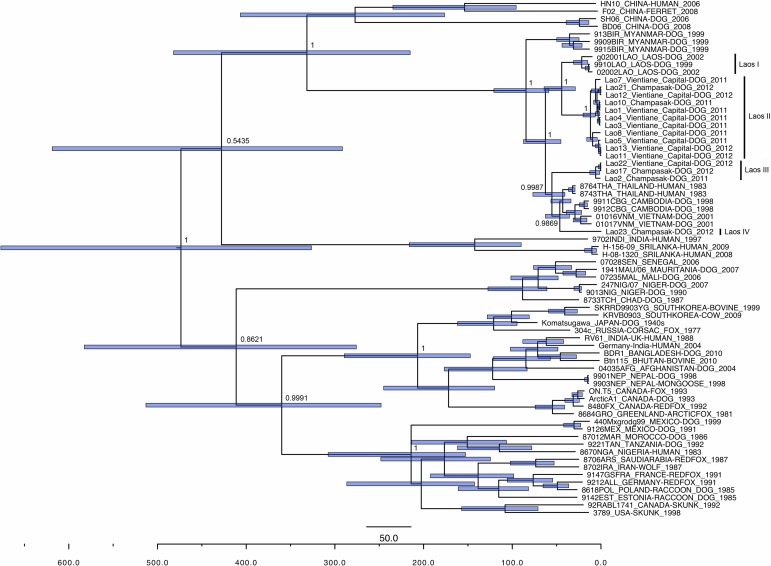
Time-line evolutionary analysis of rabies virus. Bayesian maximum credibility tree representing the genealogy of rabies virus obtained by analyzing the nucleotide sequences of the full N gene sequences (1350 nt). Rabies viruses in Laos may be divided into four lineages Lao I, II, III and IV. Nodes correspond to the mean age at which they are separated from the most recent common ancestor and blue horizontal bars at nodes represent the 95% HPDs of the most recent common ancestor. Numbers at the main nodes represent posterior values. The horizontal axis at the bottom represents time scales in years starting from the year 2012. Each strain name is followed by country of origin, host, year of detection and GenBank accession number. The nucleotide sequence data of the N gene of Lao rabies viruses appear in the DDBJ/EMBL/GenBank nucleotide sequence databases with the accession numbers: AB981665 (strain Lao1), AB981666 (strain Lao3), AB981667 (strain Lao5), AB981668 (strain Lao7), AB981669 (strain Lao8), AB981670 (strain Lao10), AB981671 (strain Lao11), AB981672 (strain Lao12), AB981673 (strain Lao13), AB981674 (strain Lao17), AB981675 (strain Lao21), AB981676 (strain Lao22) and AB981677 (strain Lao23).

Phylogenetic tree (Fig 2) revealed that rabies strains in Laos belong to the Southeast Asian (SEA) cluster that diverged from the Chinese strains approximately 331.5 years (95% HPD 215.1–481.9 years) ago. From the most recent common ancestor (TMRCA) of SEA cluster, approximately 84.5 years (95% HPD 58.8–120.7 years) ago, i.e., around 1928 (95% HPD range 1891 to 1953), rabies virus of Myanmar segregated. Approximately 62.6 years (95% HPD 45.1–87.4 years) ago, i.e., around 1949 (95% HPD range 1925 to 1967), a cluster only containing Laos rabies viruses segregated from TMRCA of the rest of the SEA cluster which contained viruses from Laos, Cambodia, Vietnam and Thailand. The Lao rabies viruses segregated approximately 44.2 years (95% HPD 29.0–64.1 years) ago, i.e., around 1968 (95% HPD range 1948 to 1983), into two lineages, one containing viruses circulated during 1999–2002 designated as Laos I, and the other contained viruses we identified in 2011 and 2012 designated as Laos II. This lineage had 11 viruses only 2 were from Champasak district and rest from Vientiane capital. Approximately 55.3 years (95% HPD 40.7–76.7 years) ago i. e. in 1957 (95% HPD 1935–1971) Laos III, containing one rabies virus from Vientiane capital and two from Champasak, diverged from rest of SEA cluster which still contained a rabies viruses from Laos. We designated this lineage as Laos IV which segregated from the other lineages of the SEA cluster, only containing rabies viruses from Cambodia, Vietnam and Thailand, approximately 46.4 years (95% HPD 35.6–62.7 years) ago, i.e. in 1966 (95% HPD 1949–1976). All lineages were supported by significant posterior value.

### Molecular analysis of rabies virus strains

We completed the whole genome sequence of two strains, strains Lao2 and Lao4 belong to Lao II and Lao III lineage, respectively. The full genome of both strains was 11924 nt long. At whole genome level there was 97.2% identity between the strains. The N, P, M, G and L genes, and G–L intergenic region were of same length (Table 3). The nucleotide identity of the genes and their deduced amino acid identity were 96.2–97.6% and 97.6–99.6%, respectively (Table 3). To further identify the different between Lao II and Lao III lineages, strain Lao2 were compared with strain Lao4 to detect the substitutions in the deduced amino acid sequences (Table 4). A total of 33 substitutions were identified among them 14 were in various domains of the proteins and 19 were in portion of the protein where no site/domain/region has been identified.

**Table 3:** The nucleotide lengths of all genes and their deduced amino acid sequences of rabies viruses from Laos are shown.

Gene	Nucleotide Length	% nucleotide identity	Amino Acid Length	% Amino Acid Identity
Genome	11924	97.2		
N	1353	97.6	450	99.6
P	894	97.1	297	98.0
M	639	96.2	212	97.6
G	1575	97.6	524	98.7
G-L region	516	95.9		
L	6387	97.4	2128	99.4

The nucleotide and amino acid identities of different genes between Lao2 and Lao4 are also shown.

**Table 4:** Substitutions in genome sequence of rabies virus strains Lao2 and Lao4 from Laos.

Protein	Lao2	Lao4	Site/domain/region of protein
N	Ser_84_	Ala_84_	
	Val_281_	Ile_281_	
P	Val_73_	Ala_73_	N protein binging site, Variable domain I
	Lys_76_	Gln_76_	N protein binging site, Variable domain I
	Ser_141_	Leu_141_	N protein binging site, Variable domain II
	Ala_149_	Ser_149_	N protein binging site, Variable domain II
	Ser_157_	Leu_157_	N protein binging site, Variable domain II
	Ile_257_	Leu_257_	
M	Gly_9_	Asp_9_	
	Glu_10_	Lys_10_	
	Ser_30_	Phe_30_	
	Ala_34_	Thr_34_	
	Val_105_	Ile_105_	
G	Ala_249_	Val_249_	Transmembrane domain
	Thr_293_	Ser_293_	Transmembrane domain
	Thr_454_	Met_454_	Cytoplasmic domain
	Thr_458_	Met_458_	Cytoplasmic domain
	Thr_464_	Ala_464_	Cytoplasmic domain
	Gly_468_	Glu_468_	
	Pro_474_	Leu_474_	
L	Ser_25_	Ala_25_	Conserved domain I
	Lys_381_	Arg_381_	Conserved domain III
	Lys_432_	Arg_432_	Conserved domain III
	Asn_675_	Asp_675_	Conserved domain V
	Asp_684_	Asn_684_	
	Pro_1093_	Ser_1093_	
	Arg_1626_	Lys_1626_	
	Ile_1659_	Val_1659_	
	Glu_1840_	Asp_1840_	
	Val_1861_	Met_1861_	
	Ala_1883_	Thr_1883_	
	Thr_2090_	Ile_2090_	
	Gln_2105_	Arg_2105_	

### Whole genome phylogeny

**Fig 3:**
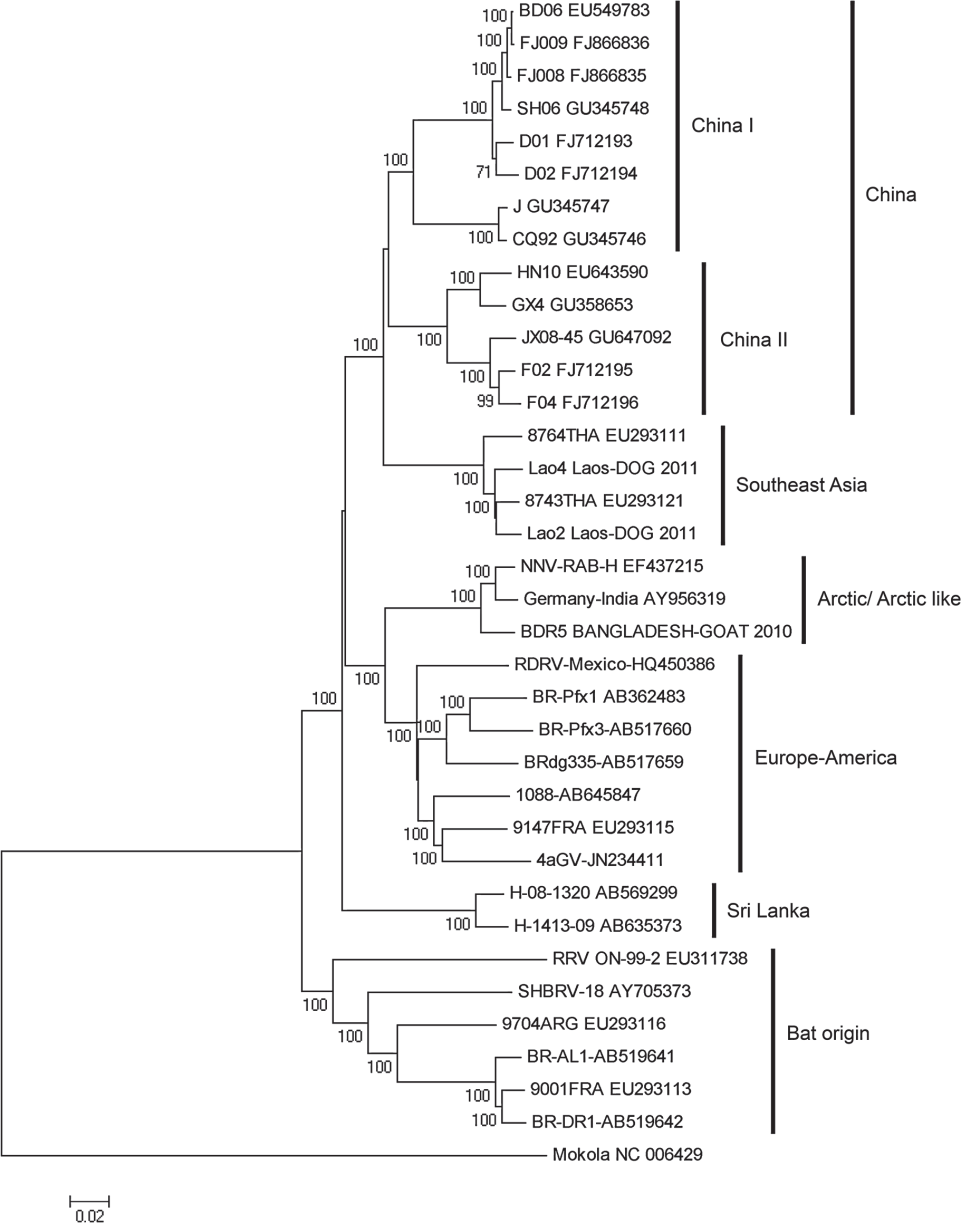
Whole genome phylogenetic tree of rabies virus. Phylogenic tree constructed with the nucleotide sequence of the whole genome of strains Lao2 belongs to lineage III and Lao4 belongs to lineage II from Laos PDR and strains from different countries available in the GenBank. Mokola was used as an out-group. Based on the whole genome phylogenetic analysis rabies viruses may be divided into China, Southeast Asia, Arctic/acrtic-like. Euro-America, Sri Lanka and Bat origin clusters. Laos rabies viruses belongs to the Southeast Asian cluster with strains from Thailand. The number adjacent to the node represents the bootstrap value and values lower than 50% have not been indicated. Scale bar shows genetic distance expressed as amino acid substitutions per site. The DNA Data Bank of Japan/European Molecular Biology Laboratory/GenBank Accession numbers of each strain described following, Lao2 and Lao4: AB981663 and AB981664.

Phylogenetic tree constructed by using whole genome showed that the bat origin rabies viruses from Americas formed a separate cluster from rest of the strains (Fig 3). Rest of the strains was divided into China, Southeast Asia, arctic/arctic like, Europe-America and Sri Lanka clusters. The China cluster consists of two major sub-clusters China I and China II. All clusters and sub-clusters were supported by bootstrap value of 100. Phylogenetic tree revealed that rabies strains from Laos belong to the SEA cluster with Thai strains. Strain Lao4 from Champasak province was closer to Thai strain than strain Lao2 from Vientiane capital.

## Discussion

In Laos, a National Committee for Rabies Control was formed in 1999 and ASEAN has also called for action to eliminate rabies by 2020 from South East Asia [12]. Therefore there have been continued activities such as attempt to increase rabies awareness among people, campaign for animal and human vaccines, and campaign for sterilizing roaming dogs. Whether the gradual increase of rabies samples as noted in this study indicating an increase of rabies in dogs or rabies awareness among the people is not known. If this indicates an increase of rabies then it needs urgent measures to contain the situation before it become big. In Laos annual number of humans with animal bite has increased from 8,277 in 2008 to 14,156 in 2011, (Expanded Program on Immunization unit, Mother and Child Health Centre, Hygiene and Health Promotion Department, Ministry of Health, Lao PDR). Vientiane Capital accounts for 73% of the national data for bites and rabies vaccination in humans, 99% of the bite victims are given postexposure treatment but in the form of vaccination only since rabies immunoglobulin is not available and only 30% receive a full course of vaccine [12]. Dog vaccination coverage was also found to be low, only 11% of the owned dogs were vaccinated for rabies [13]. Other study showed that 71–83% of dog vaccination occurs in the capital, covering 6% of the estimated dog population [12]. All samples used in the present study for genetic characterizations of rabies viruses were from owned dogs but none of them were vaccinated, over that no owner restricted the dog movements at all times through fencing, leashing or confined to indoors. The low rate of vaccination might be multifactorial and one of them may be cost. Government sponsored campaign is done once a year and mainly in the big cities therefore it does not have an impact on the rural population where the burden of rabies is possibly more. Although on the campaign day the vaccine is free but other than that the dog owner has to pay for the vaccination which may cost between 20,000 and 50,000 Kip (approximately between 2.5 and 6.2 US$). Despite the inadequate level of human and animal vaccination why the reported number of human death from rabies remains at the level of 0–1 per year deserve further investigation. Rabies epidemiology depends on human- and dog-population densities as well as to the cultural and socioeconomic environments [14]. For rabies control public education on dog vaccination and responsible dog ownership is essential.

We found in this study that more than 99% of the submitted samples were from dogs and equal proportion of dog samples were likewise positive for rabies virus indicating that similar to any rabies endemic countries of Asia and Africa, in Laos also the main transmitter of rabies is dogs. However contrary to several studies which found that rabid cats pose a significant public health risk next to dogs [15–17], in Laos it seems that a relatively lower number of cats are affected by rabies. More importantly the number of samples submitted from other animals and their proportion of rabies virus positive samples were even less than cats. Why such a small number of samples are submitted from animals other than dogs is unknown, as a whole it is thought that less number of samples is received for animal rabies diagnosis in Laos. Multiple factors might be responsible for this, such as the inconveniences associated with transportation of samples from remote areas to district livestock and fisheries offices, people are unaware of rabies in other animals, and the cost of diagnosis. The provincial livestock and fisheries offices receive samples from respective district offices and send samples to the central laboratory in Vientiane capital for the diagnosis of rabies. Any disruption of this transportation process might have an adverse effect on the quality of sample. Therefore for surveillance which can reveal the real situation of rabies in Laos these hindrances should be removed.

Our data analysis revealed that Vientiane capital and Champasak province have the major share of submitted samples. Therefore we performed the genetic characterizations of rabies viruses present in the samples from these areas. It is not known why these two provinces have the major share of submitted samples, we speculate that high population density of these two provinces and ease of transport of submitted samples might be responsible for this. Among all the provinces in Laos the population density is highest in Vientiane capital followed by Champasak province[18], that might be responsible for higher number of dogs in these provinces [19] as a result higher number of samples. The phylogenetic tree of N genes showed that the epidemiology of Laos is complex, a particular province of Laos cannot be represented by a lineage, possibly the geographical distribution of different lineages of rabies viruses in this country is mosaic in nature. This is not unusual, co-circulation of two types of canine rabies virus has been documented in countries of Africa [4,20], six lineages in China [21,22] and Romania [23], and up to 30 different lineages of bat rabies virus in the United States [24]. In smaller countries possibly circulation of one lineage of rabies virus is usual [17,25]. Considering the size of the country and movement of people and dogs to and from the rabies endemic countries, it is likely that that multiple lineages will co-circulate in Laos. Similar conditions exist in neighboring Thailand where six lineages have been identified [26].

Our phylogenetic analyses were in agreement with previous studies [7,11,22,25] and revealed that rabies viruses from Thailand, Malaysia, Cambodia, Laos and Vietnam had originated from a common ancestor. The lineages circulating in Laos are closely related but are not same as in other SEA countries, which are indicating that invasion of rabies viruses in Laos from these countries possibly did not occur recently. The time-line evolutionary analysis showed that among the currently circulating lineages, Laos II lineage is the oldest followed by Laos III and Laos IV. Possibly there were several clonal expansions of the rabies viruses in Laos leading to the formation of lineages and through periodic sweeps, such as Laos I lineage is no longer seen now, shaping up the circulating rabies viruses in Laos. Several amino acid substitutions identified in strains of Lao lineages also supports that genomic modifications occurred after the introduction of rabies virus strains in Laos. The selective advantages resulted by these genomic modifications needs to be addressed in future studies.

A recent study showed that in Vietnam there has been a constant influx of rabies viruses from China or vice versa [27]. Both countries are neighbor of Laos and consume dog meat. Periodic dissemination of viruses from one region to other by regional movement of dogs possibly for meat consumption and subsequent evolution is possibly creating the related lineages of rabies viruses in SEA cluster. Exploring how dog meat consumption is affecting the movements of dogs might shed light on the evolution of rabies viruses in this region.

We did not find any substitutions in the G genes that are responsible for increase the virulence of rabies virus [28,29], though this analysis could be done in two strains only where full genome sequence were done. The phylogenetic tree generated from whole genome sequences clearly showed the distinctness of SEA branch from other branches. Complete genome sequences of rabies viruses from other SEA countries will help to understand the complexity and dissemination of rabies virus in this region.

The limitation of the study is that samples could not be collected from all provinces of Laos yet it includes two of the provinces with highest rabies burden. This work provides insight into the evolution of rabies viruses in a geographic area where genetic analyses are sparsely reported. Small regional studies of the molecular epidemiology of rabies in Laos are of limited interest and will have to be followed after more is known of the prevalence of this disease throughout the country. Data on submitted samples clearly showed that it would take substantial amount of time to collect samples from other parts of Laos. We are collecting samples from other provinces of Laos; in future it might provide more information on the distribution, spread and evolution of rabies viruses in this region.

### Conclusions

There is an increasing trend of rabies virus detection in animal samples in Laos, which may indicate increase of rabies since dog immunization rate is low. An integrated approach is needed such as public education, access to vaccine and rabies test at affordable cost and time. Since dogs are main animal reservoir of rabies virus in Laos, therefore it might be easier to control rabies if appropriate program is introduced. Circulating rabies viruses in Laos are closely related with the rabies viruses from neighboring countries. Possibly there is no recent invasion of rabies viruses in Laos but in the past there was periodic exchange of rabies viruses with neighboring countries. Dog movements through Laos for regional dog meat consumption did not cause any spillover recently. More regional laboratories should be established in different parts of the country for proper surveillance of rabies in Laos.

## Supporting Information

S1 Table Information on dog samples collected for the molecular analysis of rabies virus. Codes of corresponding districts in Fig 1 are also shown in the district column.
(DOCX)

## References

[1] Wilde H, Hemachudha T, Jackson AC (2008) Viewpoint: Management of human rabies. Trans Royal Soc Trop Med Hyg 102: 979–982. 10.1016/j.trstmh.2008.04.008 18486168

[2] Anonymous (2013) WHO Expert Consultation on Rabies. Second report. In: WHO Technical Report Series 982. World Health Organization, Geneva. 24069724

[3] Anonymous (2013) The south-east asia dog rabies elimination strategy. In: South-East Asia Rabies Strategy. Availabe: http://www.rr-asia.oie.int/fileadmin/SRR_Activities/SEA_Rabies_Strategy_-_OIE_Final_Draft.pdf. Accessed 8 March 2015.

[4] Bourhy H, Reynes JM, Dunham EJ, Dacheux L, Larrous F, et al (2008) The origin and phylogeography of dog rabies virus. J Gen Virol 89: 2673–2681. 10.1099/vir.0.2008/003913-0 18931062 PMC3326349

[5] Meng S, Sun Y, Wu X, Tang J, Xu G, et al (2011) Evolutionary dynamics of rabies viruses highlights the importance of China rabies transmission in Asia. Virol 410: 403–409. 10.1016/j.virol.2010.12.011 21195445

[6] Wertheim HF, Nguyen TQ, Nguyen KA, de Jong MD, Taylor WR, et al (2009) Furious rabies after an atypical exposure. PLoS Med 6: e44 10.1371/journal.pmed.1000044 19296718 PMC2656546

[7] Matsumoto T, Ahmed K, Wimalaratne O, Yamada K, Nanayakkara S, et al (2011) Whole-genome analysis of a human rabies virus from Sri Lanka. Arch Virol 156: 659–669. 10.1007/s00705-010-0905-8 21298456

[8] Mitui MT, Tabib SM, Matsumoto T, Khanam W, Ahmed S, et al (2012) Detection of human bocavirus in the cerebrospinal fluid of children with encephalitis. Clin Infect Dis 54: 964–967. 10.1093/cid/cir957 22238160

[9] Drummond AJ, Rambaut A (2007) BEAST: Bayesian evolutionary analysis by sampling trees. BMC Evol Biol 7: 214 17996036 10.1186/1471-2148-7-214 PMC2247476

[10] Posada D, Crandall KA (1998) MODELTEST: testing the model of DNA substitution. Bioinformatics 14: 817–818. 9918953 10.1093/bioinformatics/14.9.817

[11] Matsumoto T, Ahmed K, Wimalaratne O, Nanayakkara S, Perera D, et al (2011) Novel sylvatic rabies virus variant in endangered golden palm civet, Sri Lanka. Emerg Infect Dis 17: 2346–2349. 10.3201/eid1712.110811 22172202 PMC3311185

[12] Kamsing A, Nasipaseuth P, Archkhawong S, Southalack K, Theppangna W, et al. (2012) A review of rabies surveillance and response activities in Lao PDR to 2011. In: 15th International Congress on Infectious Diseases. Bangkok, Thailand. Abstract No: 57.014.

[13] Drew A, Fevre SJ, Stiles E, Soulivongsa L, Chittavong M, et al (7–21 October 2011) In: Preliminary report: Laos VEVEP rabies campaign Laos: Faculty of Agriculture, National University of Laos and Veterinarians without Borders-Canada. 4 p.

[14] De Mattos CC, De Mattos CA, Loza-Rubio E, Aguilar-Setien A, Orciari LA, et al (1999) Molecular characterization of rabies virus isolates from Mexico: implications for transmission dynamics and human risk. Am J Trop Med Hyg 61: 587–597. 10548293 10.4269/ajtmh.1999.61.587

[15] Hossain M, Ahmed K, Bulbul T, Hossain S, Rahman A, et al (2012) Human rabies in rural Bangladesh. Epidemiol Infect 140: 1964–1971. 22185694 10.1017/S095026881100272X

[16] Hossain M, Bulbul T, Ahmed K, Ahmed Z, Salimuzzaman M, et al (2011) Five-year (January 2004-December 2008) surveillance on animal bite and rabies vaccine utilization in the Infectious Disease Hospital, Dhaka, Bangladesh. Vaccine 29: 1036–1040. 10.1016/j.vaccine.2010.11.052 21126605

[17] Matsumoto T, Ahmed K, Karunanayake D, Wimalaratne O, Nanayakkara S, et al (2013) Molecular epidemiology of human rabies viruses in Sri Lanka. Infect Genet Evol 18: 160–167. 10.1016/j.meegid.2013.05.018 23722023

[18] Anonymous (2015) Provinces of Laos. In: Wikipedia. Available: http://www.rr-asia.oie.int/fileadmin/SRR_Activities/SEA_Rabies_Strategy_-_OIE_Final_Draft.pdf. Accessed 8 March 2015.

[19] Hossain M, Ahmed K, Marma AS, Hossain S, Ali MA, et al (2013) A survey of the dog population in rural Bangladesh. Prev Vet Med 111: 134–138. 10.1016/j.prevetmed.2013.03.008 23590964

[20] Talbi C, Holmes EC, de Benedictis P, Faye O, Nakoune E, et al (2009) Evolutionary history and dynamics of dog rabies virus in western and central Africa. J Gen Virol 90: 783–791. 10.1099/vir.0.007765-0 19264663

[21] Tao XY, Tang Q, Rayner S, Guo ZY, Li H, et al (2013) Molecular phylodynamic analysis indicates lineage displacement occurred in Chinese rabies epidemics between 1949 to 2010. PLoS Negl Trop Dis 7: e2294 10.1371/journal.pntd.0002294 23875035 PMC3708843

[22] Gong W, Jiang Y, Za Y, Zeng Z, Shao M, et al (2010) Temporal and spatial dynamics of rabies viruses in China and Southeast Asia. Virus Res 150: 111–118. 10.1016/j.virusres.2010.02.019 20214936

[23] Turcitu MA, Barboi G, Vuta V, Mihai I, Boncea D, et al (2010) Molecular epidemiology of rabies virus in Romania provides evidence for a high degree of heterogeneity and virus diversity. Virus Res 150: 28–33. 10.1016/j.virusres.2010.02.008 20178821

[24] Hayman DT, Bowen RA, Cryan PM, McCracken GF, O'Shea TJ, et al (2013) Ecology of zoonotic infectious diseases in bats: current knowledge and future directions. Zoonoses Public Health 60: 2–21. 10.1111/zph.12000 22958281 PMC3600532

[25] Jamil KM, Ahmed K, Hossain M, Matsumoto T, Ali MA, et al (2012) Arctic-like rabies virus, Bangladesh. Emerg Infect Dis 18: 2021–2024. 10.3201/eid1812.120061 23171512 PMC3557895

[26] Lumlertdacha B, Wacharapluesadee S, Denduangboripant J, Ruankaew N, Hoonsuwan W, et al (2006) Complex genetic structure of the rabies virus in Bangkok and its surrounding provinces, Thailand: implications for canine rabies control. Trans Royal Soc Trop Med Hyg 100: 276–281. 16352324 10.1016/j.trstmh.2005.01.009

[27] Nguyen AK, Nguyen DV, Ngo GC, Nguyen TT, Inoue S, et al (2011) Molecular epidemiology of rabies virus in Vietnam (2006–2009). Jpn J Infect Dis 64: 391–396. 21937820

[28] Badrane H, Bahloul C, Perrin P, Tordo N (2001) Evidence of two Lyssavirus phylogroups with distinct pathogenicity and immunogenicity. J Virol 75: 3268–3276. 11238853 10.1128/JVI.75.7.3268-3276.2001 PMC114120

[29] Takayama-Ito M, Ito N, Yamada K, Sugiyama M, Minamoto N (2006) Multiple amino acids in the glycoprotein of rabies virus are responsible for pathogenicity in adult mice. Virus Res 115: 169–175. 16188341 10.1016/j.virusres.2005.08.004

