# Supplementary material for: Molecular Epidemiology of Rabies Viruses Circulating in Two Rabies Endemic Provinces of Laos, 2011–2012: Regional Diversity in Southeast Asia
Source: PLoS Negl Trop Dis. 2015 Mar 31;9(3):e0003645. doi: 10.1371/journal.pntd.0003645 (PMC4380407; doi:10.1371/journal.pntd.0003645)
Supplement: S1 Table — Codes of corresponding districts in Fig 1 are also shown in the district column. (DOCX) [file pntd.0003645.s001.docx]

S1 Table

**Information on dog samples collected for the molecular analysis of rabies virus.**

Codes of corresponding districts in Fig 1 are also shown in the district column.

| Sample No. | Receiving Date (dd/mm/yyyy) | Gender | | Source of sample | | | | |
| --- | --- | --- | --- | --- | --- | --- | --- | --- |
|  |  |  |  | **Village** | | **District (Code)** | | **Province** |
| Lao1 | 01/11/2011 | Male | | | Sivilay | | Xaythany (VT_3) | Vientiane Capital |
| Lao2 | 04/11/2011 | Female | | | 13 KM | | Bachieng (CH-2) | Champasak |
| Lao3 | 07/11/2011 | Female | | | Donenoun | | Xaythany (VT-3) | Vientiane Capital |
| Lao4 | 18/10/2011 | Female | | | Naunekeo | | Sikhottabong (VT-5) | Vientiane Capital |
| Lao5 | 01/09/2011 | Female | | | Tadthong | | Sikhottabong (VT-5) | Vientiane Capital |
| Lao6 | 02/09/2011 | Female | | | Kaengkew | | Sanasomeboune (CH-1) | Champasak |
| Lao7 | 02/10/2011 | Male | | | Chanesavang | | Xaythany (VT-3) | Vientiane Capital |
| Lao8 | 13/06/2011 | Female | | | Nauneborkeo | | Xaythany (VT-3) | Vientiane Capital |
| Lao9 | 21/02/2011 | Female | | | Thatkhao | | Sisattanak (VT-7) | Vientiane Capital |
| Lao10 | 21/02/2011 | Male | | | Ban Thong | | Pakse (CH-3) | Champasak |
| Lao11 | 12/3/2012 | | Male | | Viengkham | | Sikhottabong (VT-5) | Vientiane Capital |
| Lao12 | 7/3/2012 | | Male | | Tanmixay | | Xaythany (VT-3) | Vientiane Capital |
| Lao13 | 2/2/2012 | | Male | | Saphanthongtai | | Sisattanak (VT-7) | Vientiane Capital |
| Lao14 | 25/1/2012 | | Male | | Khamsavath | | Saysettha (VT-8) | Vientiane Capital |
| Lao15 | 9/1/2012 | | Male | | Savang | | Hatsayfong (VT-9) | Vientiane Capital |
| Lao16 | 4/1/2012 | | Male | | Thatlouangtai | | Saysettha (VT-8) | Vientiane Capital |
| Lao17 | 17/2/2012 | | Female | | KM 40 | | Pathoumphone (CH-10) | Champasak |
| Lao18 | 13/2/2012 | | Male | | Phounsaen | | Meuan (VP-4) | Vientiane Province |
| Lao19 | 16/2/2012 | | Female | | Sikeuth | | Naxaythong (VT-2) | Vientiane Capital |
| Lao20 | 2/3/2012 | | Male | | Tanmixay | | Xaythany (VT-3) | Vientiane Capital |
| Lao21 | 4/3/2012 | | Female | | Phonhsa-ath | | Khong (CH-9) | Champasak |
| Lao22 | 7/3/2012 | | Female | | Tanmixay | | Xaythany (VT-3) | Vientiane Capital |
| Lao23 | 2/3/2012 | | Male | | Fadaeng | | Phonhthong (CH-5) | Champasak |
